# Negative associations between folate and bacterial vaginosis in the NHANES 2001 to 2004

**DOI:** 10.1186/s12879-023-08318-5

**Published:** 2023-07-19

**Authors:** Ting-Ting Cui, Jing Luo, Rui-Lan Deng, Yun-Ting Yang, Ya-Wen Yin, Xing-Fei Chen, Hao-Kai Chen, Wan-Zhe Liao, Ze-Min Huang, Xiao-Yan Deng, Xu-Guang Guo

**Affiliations:** 1 Department of Clinical Laboratory Medicine, The Third Affiliated Hospital of Guangzhou Medical University, Guangzhou, 510150 China; 2 Department of Pediatrics, The Pediatrics School of Guangzhou Medical University, Guangzhou, 511436 China; 3 Department of Clinical Medicine, The Third Clinical School of Guangzhou Medical University, Guangzhou, 511436 China; 4 KingMed School of Laboratory Medicine, Guangzhou Medical University, Guangzhou, 511436 China; 5 Department of Clinical Medicine, The Nanshan College of Guangzhou Medical University, Guangzhou, 511436 China; 6 Guangzhou Key Laboratory for Clinical Rapid Diagnosis and Early Warning of Infectious Diseases, KingMed School of Laboratory Medicine, Guangzhou Medical University, Guangzhou, 511436 China; 7 Guangdong Provincial Key Laboratory of Major Obstetric Diseases, The Third Affiliated Hospital of Guangzhou Medical University, Guangzhou, 510150 China; 8 Key Laboratory of Reproduction and Genetics of Guangdong Higher Education Institutes, The Third Affiliated Hospital of Guangzhou Medical University, Guangzhou, 510150 China

**Keywords:** Serum folate, Red blood cell (RBC) folate, Bacterial vaginosis, NHANES, Health

## Abstract

**Background:**

Bacterial vaginosis (BV) is one of the most common infections among women of reproductive age and accounts for 15–50% of infections globally. The role played by folate in the pathogenesis and progression of BV is poorly understood. The aim of this study was to investigate the association between serum folate, red blood cell (RBC) folate, and BV in American women.

**Methods:**

1,954 participants from the 2001-2004 National Health and Nutrition Examination Survey (NHANES) program were included in this study. Multiple logistic regression was used to analyze the association between serum folate, RBC folate, and BV, and covariates including race, age, education level, and body mass index were used to construct adjusted models. Stratified analysis was used to explore the stability of the above associations in different populations.

**Results:**

In the present cross-sectional study, we found that serum folate and RBC folate were inversely associated with the risk of BV. In the fully adjusted model, the risk of BV was reduced by 35% (OR=0.65, 95% CI: 0.51~0.83, p=0.0007) in the highest serum folate group and 32% (OR=0.68, 95% CI: 0.53~0.87, p=0.0023) in the highest RBC folate group compared to the lowest group.

**Conclusions:**

The results of this study indicated that serum folate and RBC folate were inversely associated with the risk of BV folate supplementation may play an important role in the prevention and management of BV.

## Introduction

Bacterial vaginosis (BV) is a syndrome caused by mixed infection of *Gardnerella vaginalis* and some anaerobic bacteria that causes an imbalance in the vaginal microbiome, resulting in increased vaginal discharge, fishy vaginal odour, itching, and burning of the vulva. Women with BV infection are at increased risk of certain sexually transmitted diseases (e.g., HIV, *Neisseria gonorrhoeae*, *Chlamydia trachomatis*, and HSV-2), complications after gynaecological surgery, pregnancy complications, and recurrence of BV [1, 2]. BV also increases the risk of HIV transmission to their sexual partners [3].

 Treatment is usually effective, but it is prone to recur after cessation of medications [4–7]. Prevalence rates of BV are varied considerable from the geographic regions of the world, within the same country, and even within the same population, ranging from 4 to 75%, depending on the population studied, with an intermediate level in the USA (29%) [8, 9].

Theoretically, susceptibility to BV may be increased by factors that compromise the immune system, including folate deficiency. An American study found that the folate antagonist methotrexate, used to treat autoimmune and chronic inflammatory diseases, inhibits thymidine synthesis and induces apoptosis of activated T lymphocytes [10]. Weinstein's studies have shown that low serum and red blood cell folate are associated with an increased risk of invasive cervical cancer, but no studies have demonstrated the effects of serum folate and RBC folate on BV [11]. Therefore, the aim of this study was to investigate the association of serum folate and folate with the risk of BV and to further explore folic acid supplementation as an intervention strategy to reduce the risk of BV.

## Materials and methods

### Data sources

**Fig. 1:**
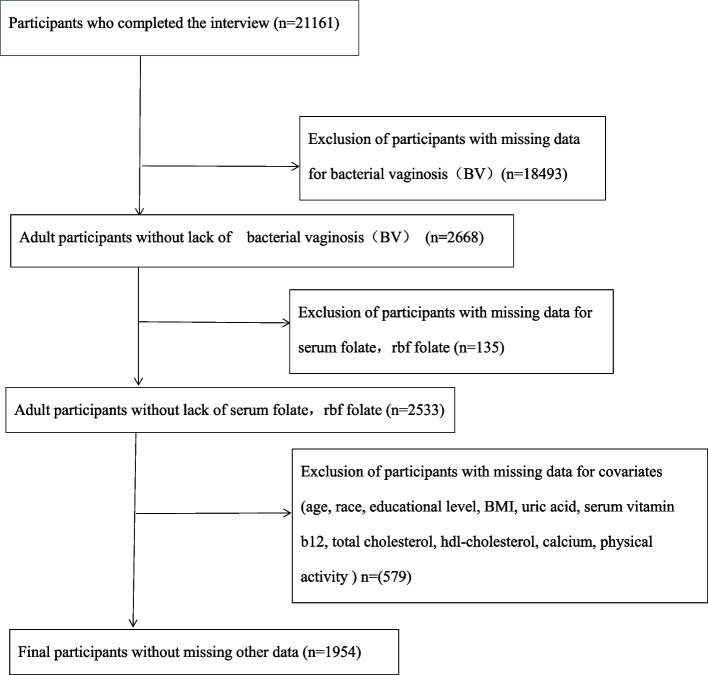
The flow chart of the study

Source of our datas were selected from The National Health and Nutrition Examination Survey (NHANES) with survey cycle from 2001-2002 and 2003-2004, a program of studies intended for assessing the health and nutritional status of noninstitutionalized U.S. citizens [12, 13]. Missing data on BV status, serum folate, and RBC folate (*n* = 18,628) were first excluded, and then other variables including age, race, education level, BMI, uric acid, serum vitamin B12, total cholesterol, HDL-cholesterol, calcium, and physical activity (*n* = 579) were dislodged, 1,954 female participants fit the selection criteria were included in our analysis. A flowchart of the screening process is depicted in Fig. 1. Datas from this study are accessible through publicly available NHANES data files.

### Determination of serum folate concentrations

The serum folate concentration was measured using the Bio-Rad Laboratories' Quantitative Phase II Folate Radioassay Kit. The method was to combine the serum sample with 125I-folate in a liquor consisting of dithiothreitol (DTT) and cyanide. The mixture was heated to collect to devitalize folate-binding protein from endogenesis. During heating, DTT stabilized reduced folate and its analogues. The mixture was cooled and bound to the affinity-purified porcine inherent factor with immobilization and folinic acid binding protein, and the pH of the reactants was adjusted until it reached 9.2. Then, the mixture was reacted at room temperature for one hour.

Endogenous folate and marked folate compete on a relative concentration basis for a limited binding site. After centrifugation and decantation, the reaction mixtures were analysed. Marked and unmarked folate bound to the immobilized protein was deposited as particles at the bottom of the test tube. Unbound folate in the supernatant was cast off, and the radioactivity from the granules was calculated. The criteria curve was drawn adopting a precalibrated folate/B12 standard in the human serum albumin base. Participants' serum folate concentrations were calculated from standard curves.

### Determination of RBC folate concentrations

Procedure for the RBC folate that samples were first diluted 1:11 with 1 g/dL ascorbic acid in an aqueous solution. Then, an incubation period of 90 min followed before the assay or immediately frozen to hemolyze RBC for subsequent assay. By both methods, the endogenous folate conjugate hydrolyzed the conjugated pteryla polyglutamate before the assay. Samples and the protein diluent, human serum albumin, were diluted 1:2 to give matrices similar to the standard and serum samples.

### Diagnosis of BV

BV measurement procedures can be found easily in NHANES documentation [14]. Self-collected vaginal swabs were received from participants aged 16–49 in the Mobile Examination Center. Subsequently, these swabs were applied to pH paper and attached to a glass slide, which was Gram-stained and evaluated by NHANES personnel at their central laboratory according to Nugent's criteria. The Nugent scoring system interprets Gram-stained vaginal smears according to the morphological type of bacteria, reflecting the overall characteristics of the vaginal flora [15]. The slides were scanned for 964 d under a microscope with a low magnification objective to locate clusters of epithelial cells. BV outcome was recognized as positive when the score was graded from 7 to 10, and a score of 0 to 6 was considered normal [15]. We omitted women without Nugent scoring system results.

### Covariates

As the results may be influenced by multiple factors, we selected participants' age, education, race, BMI, serum vitamin B12 [16], uric acid [17], total cholesterol [18, 19], lipoprotein cholesterol, calcium [19], marital status [20, 21], and physical activity as potential covariates for this study. Classification of Mexican American, other Hispanic, non-Hispanic white, non-Hispanic black, and other races (including Multi-Racial) was used to describe the race. Education was categorized into lower than high school, high school graduate, or higher than high school. BMI was calculated based on height and weight. Marital status was rated as married, widowed, divorced, separated, never married, and living with a partner. Physical activity was classified by activity level as no activity, moderate activity, vigorous activity, and both. Specific information on serum vitamin B12, uric acid, total cholesterol, lipoprotein cholesterol, and calcium content was extracted from NHANES laboratory test data.

### Statistical analysis

R Statistical Package (The R Foundation; http://www.r-project.org; version 3.6.3) and Empower Stats (www.empowerstats.net, X&Y solutions, Inc. Boston, Massachusetts) for our data analyses to perform data processing. When describing the research population, we represent continuous variables with the mean and standard deviation and classified variables with weighted percentages (%) in descriptive analysis. For statistical significance, the χ2 test and the Kruskal‒Wallis test were used separately for categorical variables and continuous variables. OR values reflect the correlation between clinical outcome and exposure [22]. To determine the relationship between serum folate and RBC folate and the incidence of BV, we conducted stratified analysis and multiple logistic regression analysis and fitted smooth curves. We calculated a 95% confidence interval. All statistical analyses in this study were statistically significant when *p* < 0.05.

## Results

### Baseline characteristics of the study participants

Two cycles of NHANES, 2001–2002 and 2003–2004, were utilized in this study. 21,161 potentially eligible participants were screened; of these, those who completed both the interview and the MEC exam were included in our study. Participants with missing data on BV (*n* = 18,493) and serum folate and RBC folate concentrations (*n* = 135) were excluded. After further excluding those with missing data on critical covariates, 1,954 participants were enrolled in our analysis. Figure 1 depicts the flowchart of the exclusion criteria. Based on BV, the descriptive characteristics of the participants are listed in Table 1. Compared to those who were negative for BV, participants with positive BV were more likely to be non-Hispanic black, never married, had lower BMI, had lower serum folate, RBC folate, HDL-cholesterol, calcium, higher uric acid, serum vitamin B12, received less than high school education, and took part in both moderate and vigorous physical activity. No statistically significant differences were detected in age or total cholesterol (*p* > 0.05).

**Table 1:** Baseline characteristics of participants (*N* = 1954)

**Characteristic**	**Bacterial vaginosis(BV)**		
	**Negative(Nugent-BV ≤ 6)**	**Positive(Nugent-BV ≥ 7)**	***P*-value**
N	1088	866	
Age (year), Mean ± SD	29.37 ± 10.23	29.52 ± 10.58	0.755
BMI (kg/m^2)^a^, Mean ± SD	27.18 ± 6.88	28.75 ± 7.69	< 0.001
Folate, serum (ng/ml), Median (Min–Max)	11.80 (2.00–689.00)	10.10 (2.10–61.40)	0.003
Folate, rbc (ng/ml rbc), Mean ± SD	282.06 ± 126.96	247.98 ± 99.34	< 0.001
Uric acid (mg/dl), Mean ± SD	4.29 ± 0.98	4.49 ± 1.11	< 0.001
Vitamin b12, serum (pg/ml), Median (Min–Max)	437.00 (106.00–4031.00)	467.00 (87.00–34,197.00)	0.005
Total cholesterol (mg/dl), Mean ± SD	189.02 ± 39.37	187.59 ± 43.38	0.215
Hdl-cholesterol (mg/dl), Mean ± SD	57.78 ± 15.35	55.36 ± 15.73	< 0.001
Calcium (mg), Median (Min–Max)	0.00 (0.00–8400.00)	0.00 (0.00–8000.00)	< 0.001
Race			< 0.001
Mexican American	262 (24.08%)	241 (27.83%)	
Other Hispanic	39 (3.58%)	39 (4.50%)	
Non-Hispanic White	544 (50.00%)	240 (27.71%)	
Non-Hispanic Black	202 (18.57%)	314 (36.26%)	
Other Race—Including Multi-Racial	41 (3.77%)	32 (3.70%)	
Education level			< 0.001
< High school	372 (34.19%)	358 (41.34%)	
High school	216 (19.85%)	198 (22.86%)	
> High school	500 (45.96%)	310 (35.80%)	
Marital			< 0.001
Married	493 (45.31%)	272 (31.41%)	
Widowed	9 (0.83%)	9 (1.04%)	
Divorced	56 (5.15%)	67 (7.74%)	
Separated	21 (1.93%)	46 (5.31%)	
Never married	442 (40.62%)	394 (45.50%)	
Living with partner	67 (6.16%)	78 (9.01%)	
Physical activity			< 0.001
No	183 (16.82%)	207 (23.90%)	
Both	305 (28.03%)	259 (29.91%)	
Moderate	226 (20.77%)	159 (18.36%)	
Vigorous	374 (34.38%)	241 (27.83%)	

^a^BMI was calculated as the body weight in kilograms divided by the square of the height in meters

*P* value: if it is a continuous variable, it is obtained by Kruskal Wallis rank sum test. If the theoretical number of counting variables is less than 10, it is obtained by Fisher exact probability test

### Association between serum folate and BV

**Fig. 2:**
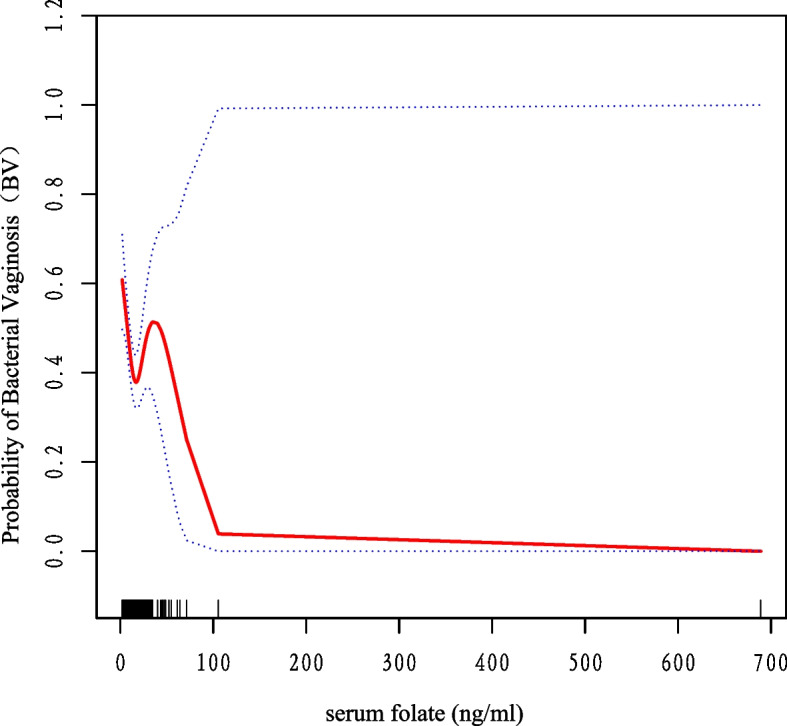
Correlation between serum folate and BV

Table 2 shows the association between the three groups of serum folate levels and BV. The crude model is an unadjusted model with no adjustments made for any variables. Adjusted model 1 was an incompletely adjusted model, adjusting mainly for demographic variables, including age, race, education level, and BMI. Adjusted model 2 was a fully adjusted model, with all included covariates adjusted. The group with the highest serum folate levels had the lowest risk of BV compared to the remaining two groups, with a 35% risk reduction in incidence compared to the low-level group, which was statistically significant (*p* = 0.0007). A negative association between serum folate levels and the incidence of BV was stable in all three models. Smoothing curves were used to visualise the association between serum folate levels and the incidence of BV, and Fig. 2 depicts the results.

**Table 2:** Association between serum folate and BV

	**Crude Model**		**Model 1**		**Model 2**	
**Outcome**	**OR (95%Cl)**	***P*-value**	**OR (95%Cl)**	***P*-value**	**OR (95%Cl)**	***P*-value**
Serum folate	0.96 (0.94, 0.97)	< 0.0001	0.98 (0.96, 0.99)	0.0041	0.98 (0.97, 1.00)	0.0460
Serum folate (trigonometry)						
T1	Reference (0)		Reference (0)		Reference (0)	
T2	0.59 (0.47, 0.74)	< 0.0001	0.68 (0.54, 0.85)	0.0009	0.69 (0.55, 0.87)	0.0020
T3	0.45 (0.36, 0.57)	< 0.0001	0.60 (0.47, 0.76)	< 0.0001	0.65 (0.51, 0.83)	0.0007
*P* for trend	< 0.0001		< 0.0001		0.0006	

Non-adjusted model adjusted for None

Model I adjusted for Age, Educational level, Race, BMI

Model II adjusted for the following: Model I + serum vitamin b12; uric acid; total cholesterol; HDL cholesterol; calcium; marital; and physical activity

### Association between RBC folate and BV

**Fig. 3:**
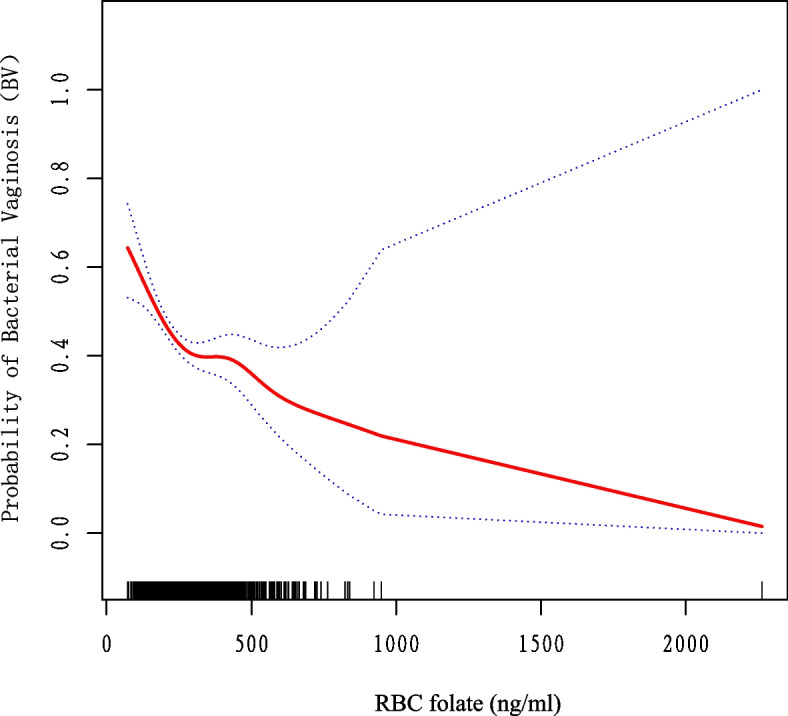
Correlation between RBC folate and BV

Table 3 shows the association between the three groups of RBC folate levels and BV, adjusting the model to be consistent with Table 2. The group with the highest RBC folate levels had the lowest risk of BV compared to the remaining two groups, with a 32% risk reduction in incidence compared to the low-level group, which was statistically significant (*p* = 0.0023) and consistent with the above results. A negative association between RBC folate levels and the incidence of BV was found to be stable in all three models. Smoothing curves were used to visualize the association between RBC folate levels and the incidence of BV, and the results are shown in Fig. 3.

**Table 3:** Association between RBC folate and BV

	**Crude Model**		**Model 1**		**Model 2**	
**Outcome**	**OR (95%Cl)**	***P*-value**	**OR (95%Cl)**	***P*-value**	**OR (95%Cl)**	***P*-value**
Rbc folate	1.00 (1.00, 1.00)	< 0.0001	1.00 (1.00, 1.00)	0.0041	1.00 (1.00, 1.00)	0.0460
Rbc folate (trigonometry)						
T1	Reference (0)		Reference (0)		Reference (0)	
T2	0.58 (0.46, 0.72)	< 0.0001	0.67 (0.53, 0.84)	0.0006	0.69 (0.55, 0.88)	0.0021
T3	0.47 (0.38, 0.59)	< 0.0001	0.61 (0.48, 0.78)	< 0.0001	0.68 (0.53, 0.87)	0.0023
*P* for trend	< 0.0001		< 0.0001		0.0019	

Non-adjusted model adjusted for None

Model I adjusted for Age, Educational level, Race, BMI

Model II adjusted for the following: Model I + serum vitamin b12; uric acid; total cholesterol; HDL cholesterol; calcium; marital; and physical activity

### Stratification analysis between serum folate and BV

**Fig. 4:**
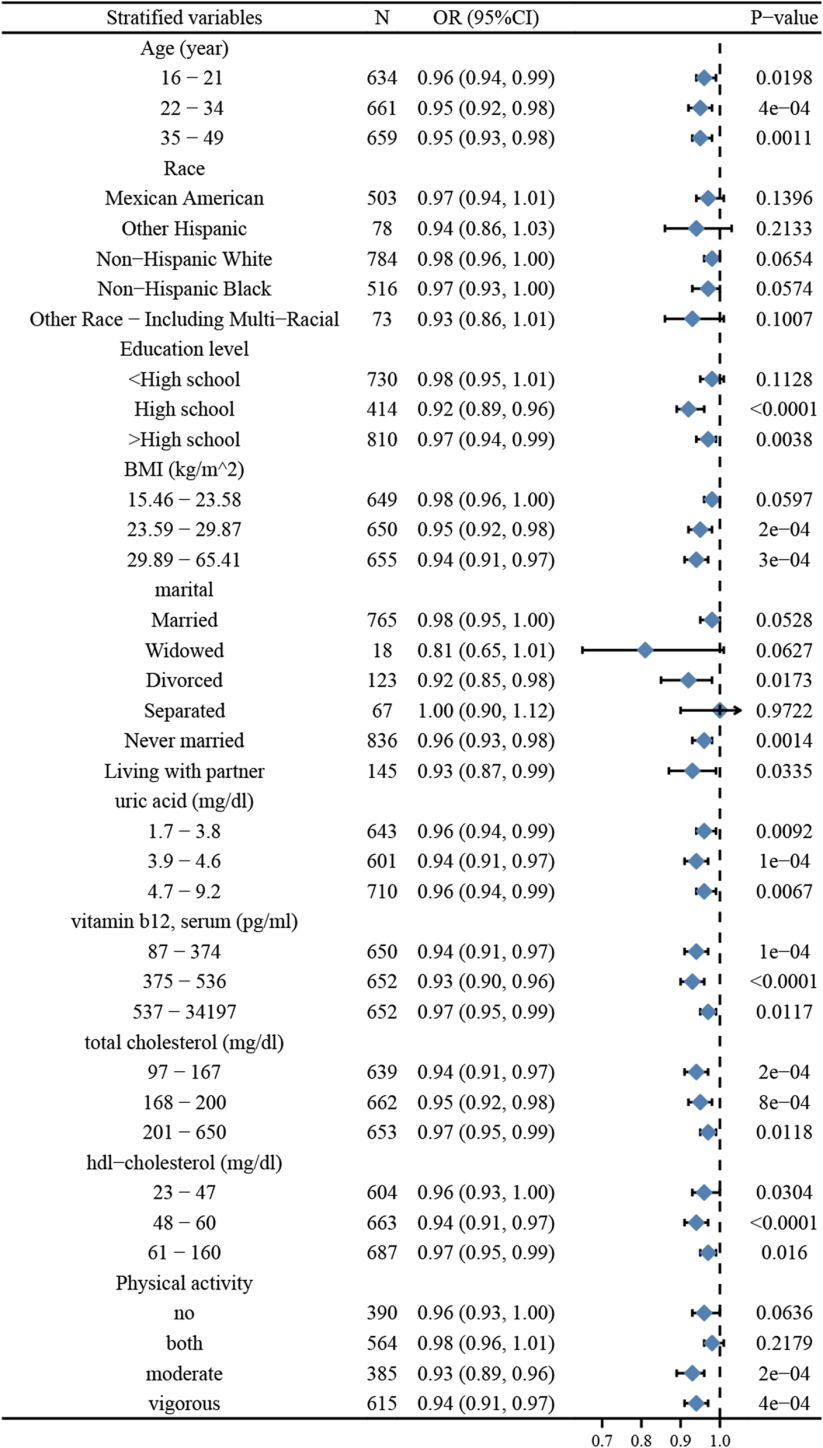
Stratification analysis between serum folate and BV

As shown in Fig. 4, stratified analyses were conducted for age, race, education level, BMI, marital status, physical activity, and relevant biochemical indicators such as uric acid, vitamin B12, HDL-cholesterol, and total cholesterol. The results of the stratified analysis showed that although the OR values fluctuated across the subgroups, the direction of the results was mostly consistent (OR < 1), indicating that the results of this study were stable and sensitive. Additionally, high heterogeneity was observed in terms of race, BMI, education, and marital status. However, it was relatively stable in the age stratification.

### Stratification analysis between RBC folate and BV

**Fig. 5:**
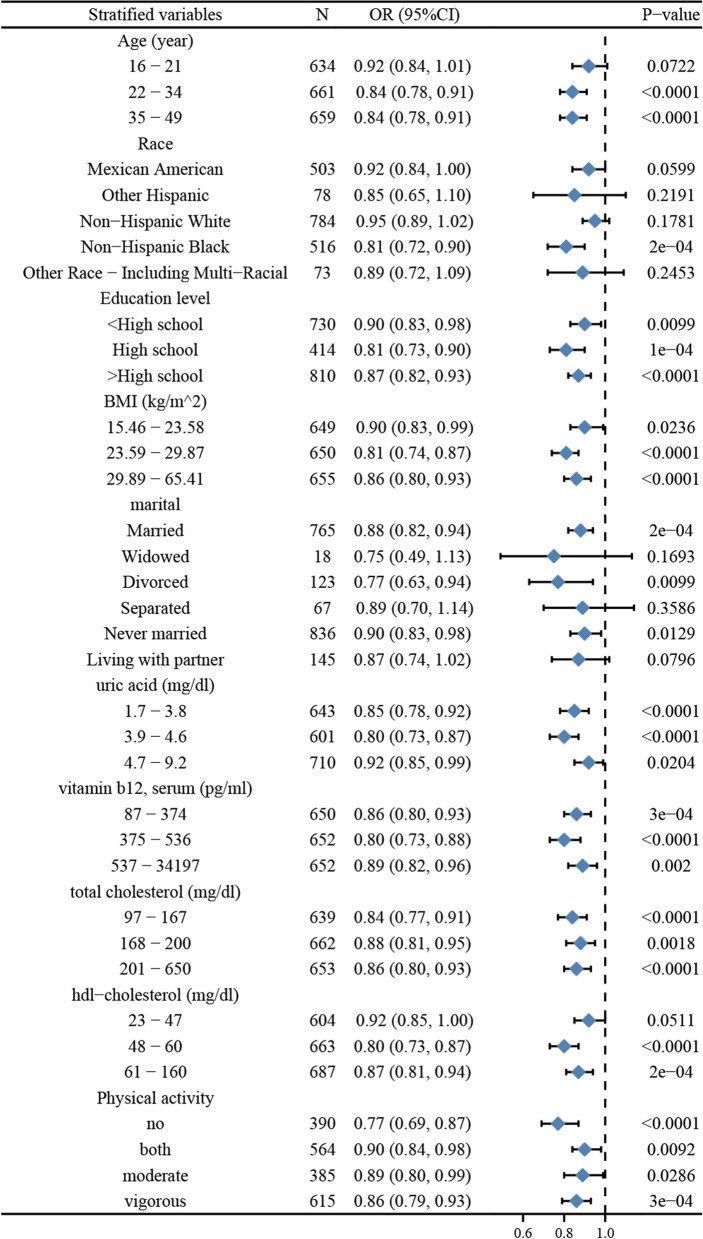
Stratification analysis between RBC folate and BV

As shown in Fig. 5, the stratified analysis revealed greater fluctuations in OR values across subgroups compared to Fig. 4. However, the directions of the results were all equally consistent (OR < 1), indicating that the results of this study remained stable and sensitive. Nevertheless, a high degree of heterogeneity was also found between the different strata of factors, and results were mostly significant.

## Discussion

The cross-sectional analysis was carried out using two consolidated datasets of NHANES surveys from 2001 to 2004. Based on the outcomes of different models, a significant negative correlation was found between RBC folate concentrations and BV, as well as between serum folate concentrations and BV.

According past researches, this is the first study is to assess the relationship between folate and BV. Only Anne L. Dunlop et al. [23] supposed that folic acid deficiency is closely related to BV during pregnancy. The results showed that with the increase in RBC folate, the incidence of BV decreased significantly. This is consistent with the research results of Neggers YH et al. [19] and Dunlop AL et al. [23], which confirmed that folate was negatively correlated with BV. The risk of BV decreased with the increase in serum folate and RBC folate, indicating that serum folate and RBC folate may affect the occurrence of BV. However, it remains unclear whether the exact mechanism of BV is affected by folic acid.

 Common general understanding that supposed immune factors may play a vital role in the pathogenesis of BV [19, 24, 25]. Studies have revealed that folic acid intake might improve immune function [26], thereby reducing the risk of severe BV [19]. Meanwhile, the capacity of folic acid is to improve indirectly mucosal immune of the female subgenital through intestinal immune regulation [27].

Mucosal immunity of the female subgenital system plays an innate immune role through neutrophils, dendritic cells, natural killer cells, and other common innate immune cells [28]. The capacity of folic acid is to maintain or enhance the cellular activity of NK cells [29]. In contrast, the role of NK cells is to enhance innate non-specific immune function, thereby improving human immune function and reducing the incidence of BV.

In addition to playing a barrier role in the innate immune response, vaginal mucosa also mediates the adaptive immune response of vaginal mucosa through IgG and secretory IgA (sIgA) [28, 30]. The ability of sIgA is to enhance the activity of neutrophils that secrete chemokines that attract macrophages, T cells, and DCs and inhibit proteolysis [31, 32]. Part of the IgG that plays an immune role is locally secreted by the vaginal mucosa, and part of it is transferred to the genital tract mucosa through the circulatory system to play a role. The role of CD8+ T cells is to promote the Th1-cell-mediated immune response to intracellular pathogens [28]. The specific immune function of folic acid is to support and promote the Th1-mediated immune response and to play a significant role in antibody production and metabolism. Its advantage is facilitating the adaptive immune response of the body and reducing the incidence of BV [29].

For the assessment of folate levels, the National Pathology Alliance benchmarking review in the UK [33] and Christopher-John L, Farrell et al. [34] recommend measurement of serum folate, whereas traditionally, studies of folate status have used serum/plasma or RBC folate, both of which are widely available in the laboratory. Among them, RBC folate is favored by many clinicians and is a more labor-intensive test [35]. Therefore, serum folate and RBC folate concentrations were used as research indicators. The results showed that serum folate and RBC folate concentrations were negatively correlated with the risk of BV, because high serum folate and RBC folate concentrations indicated high folate levels in the body, as high folate levels help to enhance the body's immune response, thereby reducing the risk of BV.

In vitro experiments in quantity and epidemiological investigations have shown that the causes of BV are very complex. No research has revealed the connection between serum or RBC folic acid and the risk of BV. Therefore, further research on the association between serum folate and RBC folate and the risk of BV will play a positive role in the treatment, prevention, and reduction of the recurrence of BV. The aim of this study was to further research the relationship between serum or RBC folic acid, and BV risk and to display the study results more comprehensively and intuitively by adjusting the methods of confounding, hierarchical analysis, multiple regression analysis, curve fitting, etc. The findings that serum or RBC folate concentrations are negatively correlated with BV provided biological rationality for the relationship between people's nutritional status and BV and could be used to guide clinical prevention, treatment, and prognosis.

However, there are limitations to the current study. First, as a cross-sectional observational study, it is difficult to determine the temporal relationship of antecedents and consequences. The results may be influenced by other unmeasured variable quantities even after multiple adjustments. Second, although many samples were used, datas were collected from 2001 to 2004. Therefore, when extrapolating to today's environment, there may be some bias It is necessary to consider biases caused by the passage of time.

## Conclusions

In conclusion, our study show that both serum folate and RBC folate are negatively associated with BV. Increasing the amount of serum folate and RBC folate in the body may boost our immune function and thereby reduce the risk of BV.

## Data Availability

Original data generated and analyzed during this study are included in this published article or in the data repositories listed in References. The dataset supporting the conclusions of this article is available in the NHANES repository, https://www.cdc.gov/nchs/nhanes/index.htm.

## References

[1] Nelson DB, Hanlon A, Hassan S, Britto J, Geifman-Holtzman O, Haggerty C, Fredricks DN (2009). Preterm labor and bacterial vaginosis-associated bacteria among urban women. J Perinat Med.

[2] Laxmi U, Agrawal S, Raghunandan C, Randhawa VS, Saili A (2012). Association of bacterial vaginosis with adverse fetomaternal outcome in women with spontaneous preterm labor: a prospective cohort study. J Matern Fetal Neonatal Med.

[3] Cherpes TL, Wiesenfeld HC, Melan MA, Kant JA, Cosentino LA, Meyn LA, Hillier SL (2006). The associations between pelvic inflammatory disease, Trichomonas vaginalis infection, and positive herpes simplex virus type 2 serology. Sex Transm Dis.

[4] Workowski KA, Bolan GA (2015). Sexually transmitted diseases treatment guidelines, 2015. MMWR Recomm Rep.

[5] AbouChacra L, Fenollar F, Diop K (2021). Bacterial Vaginosis: What Do We Currently Know?. Front Cell Infect Microbiol.

[6] Bautista CT, Wurapa E, Sateren WB, Morris S, Hollingsworth B, Sanchez JL (2016). Bacterial vaginosis: a synthesis of the literature on etiology, prevalence, risk factors, and relationship with chlamydia and gonorrhea infections. Mil Med Res.

[7] Cohen CR, Lingappa JR, Baeten JM, Ngayo MO, Spiegel CA, Hong T, Donnell D, Celum C, Kapiga S, Delany S (2012). Bacterial vaginosis associated with increased risk of female-to-male HIV-1 transmission: a prospective cohort analysis among African couples. PLoS Med.

[8] Bitew A, Abebaw Y, Bekele D, Mihret A (2017). Prevalence of Bacterial Vaginosis and Associated Risk Factors among Women Complaining of Genital Tract Infection. Int J Microbiol.

[9] Kenyon C, Colebunders R, Crucitti T (2013). The global epidemiology of bacterial vaginosis: a systematic review. Am J Obstet Gynecol.

[10] Courtemanche C, Elson-Schwab I, Mashiyama ST, Kerry N, Ames BN (2004). Folate deficiency inhibits the proliferation of primary human CD8+ T lymphocytes in vitro. J Immunol (Baltimore, Md : 1950).

[11] Weinstein SJ, Ziegler RG, Frongillo EA, Colman N, Sauberlich HE, Brinton LA, Hamman RF, Levine RS, Mallin K, Stolley PD (2001). Low serum and red blood cell folate are moderately, but nonsignificantly associated with increased risk of invasive cervical cancer in U.S. women. J Nutr.

[12] Woteki CE, Briefel RR, Kuczmarski R (1988). Federal monitoring of the nation's nutritional status. Contributions of the National Center for Health Statistics. Ame J Clin Nutr.

[13] Ahluwalia N, Dwyer J, Terry A, Moshfegh A, Johnson C (2016). Update on NHANES Dietary Data: Focus on Collection, Release, Analytical Considerations, and Uses to Inform Public Policy. Advances in nutrition (Bethesda, Md).

[14] Geller RJ, Brotman RM, O'Brien KM, Fine DM, Zota AR (2018). Phthalate exposure and odds of bacterial vaginosis among U.S. reproductive-aged women, NHANES 2001–2004. Reprod Toxicol.

[15] Nugent RP, Krohn MA, Hillier SL (1991). Reliability of diagnosing bacterial vaginosis is improved by a standardized method of gram stain interpretation. J Clin Microbiol.

[16] Yüksel H, Odabasi AR, Cetin G, Eben M, Nergiz S, Onur E (2007). Folate and vitamin B12 levels in abnormal pap smears: a case control study. Eur J Gynaecol Oncol.

[17] Jakovljević A, Bogavac M, Nikolić A, Tošić MM, Novaković Z, Stajić Z (2014). The influence of bacterial vaginosis on gestational week of the completion of delivery and biochemical markers of inflammation in the serum. Vojnosanit Pregl.

[18] Pleckaityte M (2019). Cholesterol-Dependent Cytolysins Produced by Vaginal Bacteria: Certainties and Controversies. Front Cell Infect Microbiol.

[19] Neggers YH, Nansel TR, Andrews WW, Schwebke JR, Yu KF, Goldenberg RL, Klebanoff MA (2007). Dietary intake of selected nutrients affects bacterial vaginosis in women. J Nutr.

[20] Marconi C, Duarte MT, Silva DC, Silva MG (2015). Prevalence of and risk factors for bacterial vaginosis among women of reproductive age attending cervical screening in southeastern Brazil. Int J Gynaecol Obstet.

[21] Kalinka J, Hanke W, Wasiela M, Laudański T (2002). Socioeconomic and environmental risk factors of bacterial vaginosis in early pregnancy. J Perinat Med.

[22] Andrade C (2015). Understanding relative risk, odds ratio, and related terms: as simple as it can get. J Clin Psychiatry.

[23] Dunlop AL, Taylor RN, Tangpricha V, Fortunato S, Menon R (2011). Maternal vitamin D, folate, and polyunsaturated fatty acid status and bacterial vaginosis during pregnancy. Infect Dis Obstet Gynecol.

[24] St John E, Mares D, Spear GT (2007). Bacterial vaginosis and host immunity. Curr HIV/AIDS Rep.

[25] Murphy K, Mitchell CM (2016). The Interplay of Host Immunity, Environment and the Risk of Bacterial Vaginosis and Associated Reproductive Health Outcomes. J Infect Dis.

[26] Dhur A, Galan P, Hercberg S (1991). Folate status and the immune system. Prog Food Nutr Sci.

[27] Ponziani FR, Cazzato IA, Danese S, Fagiuoli S, Gionchetti P, Annicchiarico BE, D'Aversa F, Gasbarrini A (2012). Folate in gastrointestinal health and disease. Eur Rev Med Pharmacol Sci.

[28] Reis Machado J, da Silva MV, Cavellani CL, dos Reis MA, Monteiro ML, Teixeira Vde P, Miranda Corrêa RR (2014). Mucosal immunity in the female genital tract HIV/AIDS. BioMed Res Int.

[29] Maggini S, Pierre A, Calder PC (2018). Immune Function and Micronutrient Requirements Change over the Life Course. Nutrients.

[30] Villa P, Cipolla C, D'Ippolito S, Amar ID, Shachor M, Ingravalle F, Scaldaferri F, Puca P, Di Simone N, Scambia G (2020). The interplay between immune system and microbiota in gynecological diseases: a narrative review. Eur Rev Med Pharmacol Sci.

[31] Li Y, Jin L, Chen T (2020). The Effects of Secretory IgA in the Mucosal Immune System. Biomed Res Int.

[32] Wira CR, Fahey JV, Sentman CL, Pioli PA, Shen L (2005). Innate and adaptive immunity in female genital tract: cellular responses and interactions. Immunol Rev.

[33] Galloway M, Rushworth L (2003). Red cell or serum folate? Results from the National Pathology Alliance benchmarking review. J Clin Pathol.

[34] Farrell CJ, Kirsch SH, Herrmann M (2013). Red cell or serum folate: what to do in clinical practice?. Clin Chem Lab Med.

[35] Pillay TS, Oosthuizen NM (2014). Why are we still measuring red cell folate instead of just serum folate?. J Clin Pathol.

